# Regulation of Polycystin-1 Function by Calmodulin Binding

**DOI:** 10.1371/journal.pone.0161525

**Published:** 2016-08-25

**Authors:** Nicholas Doerr, Yidi Wang, Kevin R. Kipp, Guangyi Liu, Jesse J. Benza, Vladimir Pletnev, Tengis S. Pavlov, Alexander Staruschenko, Ashraf M. Mohieldin, Maki Takahashi, Surya M. Nauli, Thomas Weimbs

**Affiliations:** 1 Department of Molecular, Cellular, and Developmental Biology and Neuroscience Research Institute, University of California Santa Barbara, Santa Barbara, CA, United States of America; 2 Department of Structural Biology, Shemyakin-Ovchinnikov Institute of Bioorganic Chemistry, Russian Academy of Sciences, Moscow, Russian Federation; 3 Department of Physiology, Medical College of Wisconsin, Milwaukee, WI, United States of America; 4 Department of Biomedical and Pharmaceutical Sciences, Chapman University School of Pharmacy, Irvine, United States of America; 5 University of California Irvine, Medical Campus, Orange, CA, United States of America; 6 Department of Nephrology, Qilu Hospital, Shandong University, Jinan, China; University of Houston, UNITED STATES

## Abstract

Autosomal Dominant Polycystic Kidney Disease (ADPKD) is a common genetic disease that leads to progressive renal cyst growth and loss of renal function, and is caused by mutations in the genes encoding polycystin-1 (PC1) and polycystin-2 (PC2), respectively. The PC1/PC2 complex localizes to primary cilia and can act as a flow-dependent calcium channel in addition to numerous other signaling functions. The exact functions of the polycystins, their regulation and the purpose of the PC1/PC2 channel are still poorly understood. PC1 is an integral membrane protein with a large extracytoplasmic N-terminal domain and a short, ~200 amino acid C-terminal cytoplasmic tail. Most proteins that interact with PC1 have been found to bind via the cytoplasmic tail. Here we report that the PC1 tail has homology to the regulatory domain of myosin heavy chain including a conserved calmodulin-binding motif. This motif binds to CaM in a calcium-dependent manner. Disruption of the CaM-binding motif in PC1 does not affect PC2 binding, cilia targeting, or signaling via heterotrimeric G-proteins or STAT3. However, disruption of CaM binding inhibits the PC1/PC2 calcium channel activity and the flow-dependent calcium response in kidney epithelial cells. Furthermore, expression of CaM-binding mutant PC1 disrupts cellular energy metabolism. These results suggest that critical functions of PC1 are regulated by its ability to sense cytosolic calcium levels via binding to CaM.

## Introduction

Autosomal Dominant Polycystic Kidney Disease (ADPKD) is a common, life-threatening genetic disease with an estimated prevalence between 1:400–1:1000 live births. The underlying causes are mutations in either the PKD1 or PKD2 genes encoding polycystin-1 (PC1) and polycystin-2 (PC2), respectively. Disease progression is characterized by renal cyst growth, grossly enlarged kidneys and eventual loss of renal function. In most patients renal failure occurs in the fourth to sixth decade of life, thus requiring lifelong dialysis or kidney transplantation [1, 2]. There is currently no FDA-approved treatment to slow disease progression. A vasopressin receptor inhibitor was recently approved for ADPKD in several countries but side effects, potential toxicity and unfavorable cost-effectiveness may limit the usefulness of this drug [3]. Therefore, there remains an urgent need for effective treatments.

PC2, also called TRPP2, is a calcium-permeable cation channel of the transient receptor potential (TRP) channel family [4]. PC1 and PC2 localize to the primary cilium and bind each other through their C-terminal coiled-coil domains suggesting that they function together as a complex. This interaction is required for calcium entry in response to apical fluid flow and bending of primary cilia [5]. In addition to calcium entry, the PC1/PC2 complex also regulates heterotrimeric G-protein signaling which depends on a G-protein activation motif in the cytoplasmic tail of PC1 [6–9]; however, it is unclear whether this regulation is dependent on calcium entry through the channel. In fact, little is known about the downstream targets of the PC1/PC2 calcium signal.

PC1 has also been found to interact with numerous other proteins and regulate downstream signaling pathways. This includes STAT3 [10, 11], STAT6 [12, 13], mTOR [14, 15], wnt signaling [16, 17], AP1 [9, 18] and others [19]. Additionally, PC1 undergoes cleavage to form several functional C-terminal fragments. Cleavage within the third intracellular loop of PC1 results in the formation of a ~100 kDa fragment that localizes to the ER and regulates ER calcium homeostasis through its interaction with STIM1 [20]. Another cleavage event produces a ~30 kDa C-terminal fragment that translocates to the nucleus and regulates the transcriptional activity of STAT3 [10, 11], STAT6 [12, 13], and β-catenin [21].

Structurally, PC1 is a large integral membrane protein with 11 transmembrane domains, a large N-terminal extracytoplasmic region that contains 16 PKD domains, an REJ domain, a C-type lectin domain, and a GPS cleavage site, as well as a ~200 residue long cytoplasmic tail [22, 23]. The cytoplasmic tail contains a coiled coil domain required for interaction with PC2 [24–26], a G-protein activation domain [7], which overlaps with a nuclear localization signal for the cleaved PC1 tail [27], and a PEST sequence [12]. The structure of specific PC1 domains has been investigated experimentally and computationally, including the PKD domains [28] and the C-type lectin domain [29], however no structural information of the cytoplasmic tail is yet available.

In this work we find that the membrane-proximal region of the PC1 cytoplasmic tail has predicted structural homology to the regulatory domain of myosin heavy chains that is known to interact with calmodulin (CaM) or the CaM-related regulatory myosin light chains. The structural model of this region includes conserved, predicted CaM binding sites, the conserved G-protein activation domain and the nuclear localization signal of the PC1 tail. We confirm the ability of PC1 to interact with CaM *in vitro* in a Ca^2+^-regulated manner. Mutation of the main CaM binding site disrupts the CaM interaction and leads to impaired Ca^2+^ channel activity of the PC1/PC2 complex, and loss of flow/cilia-mediated intracellular Ca^2+^ signaling. Disruption of the CaM binding site also leads to altered PC1-dependent regulation of cellular metabolism. These results suggest that Ca^2+^/CaM plays an important role in regulating the function of the PC1/PC2 complex.

## Results

### PC1 has homology to the regulatory domain of myosin heavy chains and is predicted to bind calmodulin

**Fig 1:**
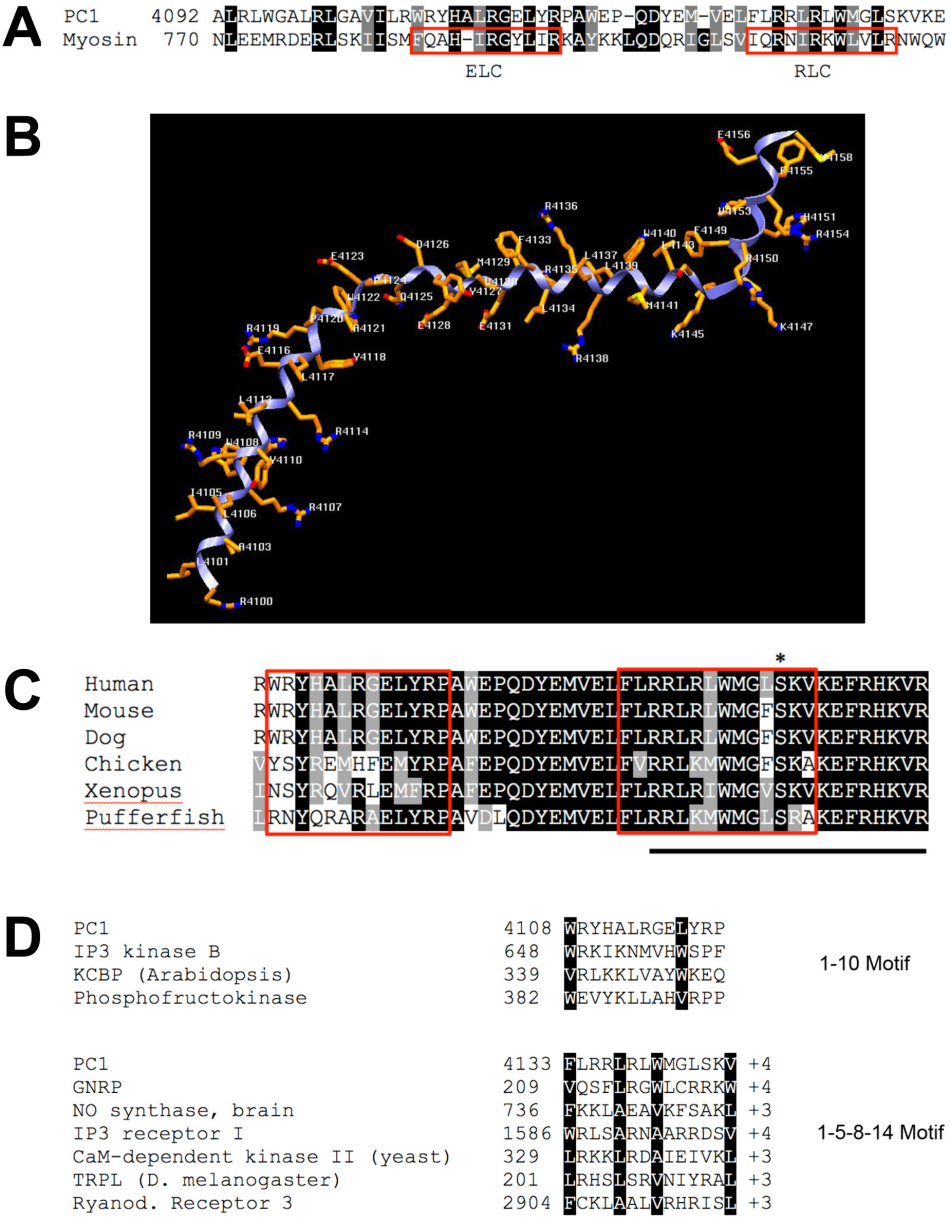
The PC1 tail has predicted homology to scallop myosin. (A) Alignment of amino acids 4092–4148 of the PC1 tail with the regulatory domain of scallop myosin heavy chain (Argopecten irradians, 1S5G_A). The two outlined myosin sequences identify the two IQ motifs that bind myosin light chains (ELC or RLC). (B) A structural model of the PC1 tail based on the known structure of scallop myosin. (C) Alignment of the human PC1 sequence with known orthologs (Mus musculus, NP_038658; Canis lupus familiaris, NP_001006651; Gallus gallus, XP_015149930; Xenopus tropicalis, XP_012826626; Takifugu rubripes, XP_011610747). The two IQ-like motifs are boxed in red, the previously identified G-protein activation sequence is underlined, and the mutated serine that results in attenuated CaM binding is identified with an asterisk. (D) Alignment of the two putative CaM binding domains in PC1 with proteins containing known CaM binding motifs (NP_002212.3, NP_851276, NP_001160158, NP_002882.3, NP_000611.1, NP_001093422.2, NP_014626, NP_476895, NP_001027.3). Hydrophobic amino acids at characteristic positions within each sequence are highlighted. The first predicted CaM binding domain in PC1 (above) best resembles a 1–10 motif, with bulky hydrophobic residues at positions 1 and 10 in the sequence. The second predicted domain (below) resembles a 1-5-8-14 motif, with bulky hydrophobic residues at these positions and a characteristic ≥+3 charge.

Most of the reported protein interactions with PC1 occur with its ~200 amino acid cytoplasmic tail [19], however little is known about the structure of this domain. To learn more about the putative structure of the PC1 tail, we performed a homology search against proteins with known structural information in the Protein Data Bank (www.rcsb.org [30]). Pairwise alignments led to the identification of a portion of the PC1 tail (residues 4100–4143) with significant homology to the regulatory domain of scallop myosin (Fig 1A).

This region of myosin heavy chains from many species typically contains two tandem IQ motifs that form binding sites for CaM or the CaM-related proteins myosin essential light chain (ELC) and myosin regulatory light chain (RLC), respectively [31–33]. An extended sequence comparison between PC1 and myosin heavy chains from a variety of divergent species shows that while PC1 is clearly distinct from myosins, some of the most conserved myosin residues are also conserved in PC1 (S1 Fig).

Based on the primary sequence alignment and the known 3D-structure of scallop myosin in complex with ELC and RLC [31], we modeled the predicted structure of the corresponding region of the PC1 tail which resulted in a relatively extended, S-shaped alpha helix (Fig 1B).

Given this putative structural homology, we hypothesized that this region of PC1 may interact with CaM. The PC1 tail sequence was analyzed for possible CaM binding motifs using a prediction algorithm (Calmodulin Target Database) [34, 35]. Indeed the algorithm predicts two CaM binding motifs that overlap with the regions of homology to the ELC and RLC binding motifs in myosin heavy chain (Fig 1C). However, PC1 does not contain a classical IQ motif as it lacks the obligate glutamines in the second position. Instead, the first putative CaM binding site conforms to a “1–10” CaM binding motif, whereas the second motif conforms to a “1-5-8-14” CaM binding motif (Fig 1D). These motifs are named for the placement of bulky hydrophobic residues within a CaM target sequence [34, 36]. Additionally, 1-5-8-14 motifs also tend to carry a net positive charge of +3 or greater. Unlike IQ motifs that classically bind CaM in the absence of Ca^2+^, these two motifs typically bind CaM only in the presence of Ca^2+^.

This region of PC1 is well conserved among PC1 orthologs, with higher conservation of the second putative CaM binding motif than the first motif (Fig 1C). The second predicted CaM-binding domain also overlaps with the previously identified G-protein activation domain [7], highlighting the importance of this region to the function of PC1.

### The PC1 tail interacts with Ca^2+^/CaM

**Fig 2:**
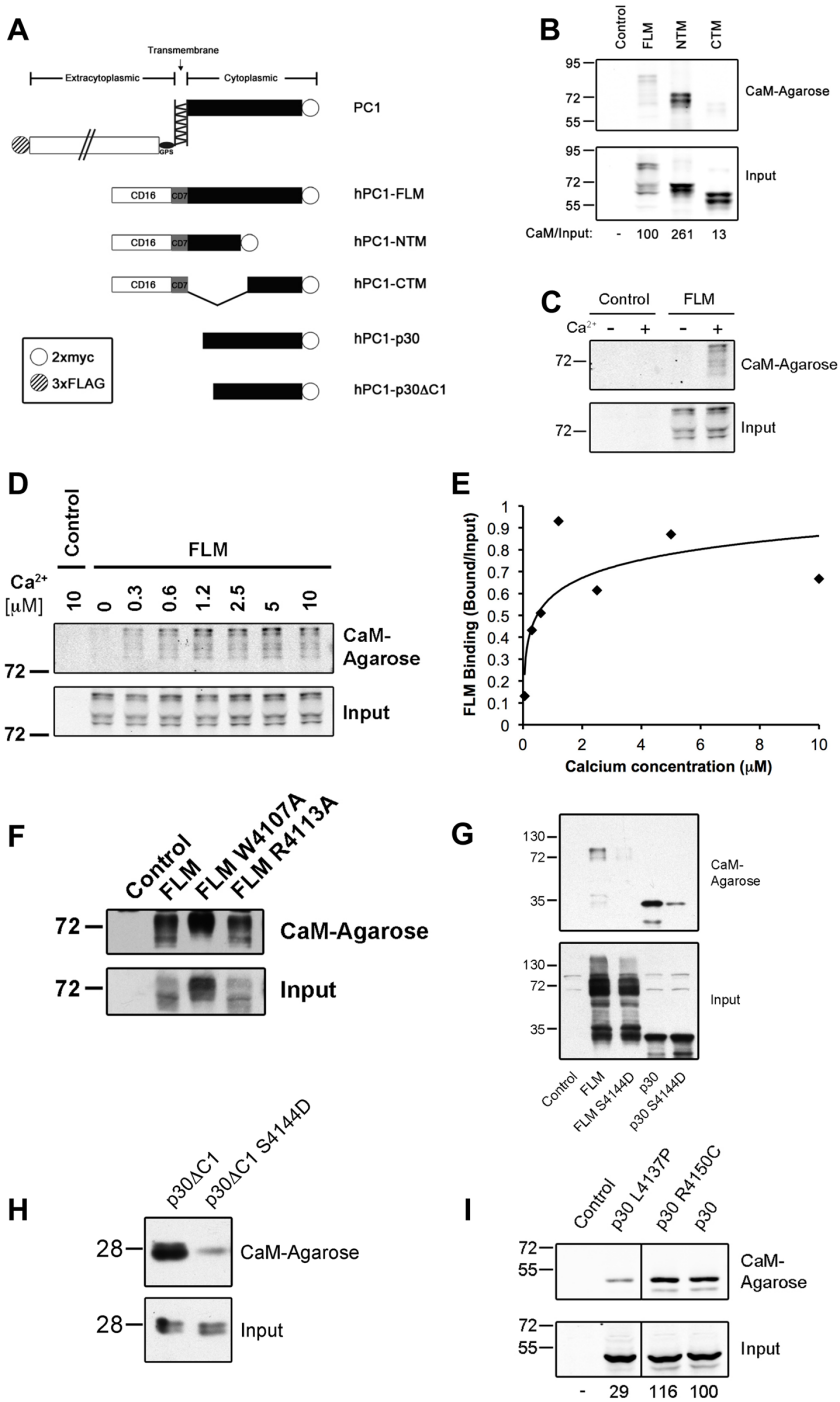
Calmodulin binds the PC1 tail in the presence of calcium. (A) Map of PC1-expression constructs: Full-length membrane-anchored (FLM), N-terminal membrane-anchored (NTM), C-terminal membrane-anchored (CTM), the soluble tail (p30), and fragment that completely lacks the first putative CaM-binding motif (p30dC1). (B) FLM, NTM, or CTM were precipitated from lysates of transiently expressing HEK293T cells with CaM-agarose beads and analyzed by immunoblotting. (C) Binding of PC1-FLM to CaM was analyzed in the presence or absence of 200 μM buffered calcium. (D-E) Binding experiment performed in the presence of a range of buffered calcium concentrations. (F) Binding experiment using indicated mutants within the first putative CaM-binding motif. (G) Binding experiment using either FLM or p30 with the indicated mutation within the second putative CaM-binding motif. (H) Binding experiment performed with a wild-type or mutant p30dC1 PC1 fragment. (I) Binding experiment performed using wild-type or patient mutants of PC1-p30.

The data from these in silico analyses led us to hypothesize that the PC1 tail may interact with calmodulin in the presence of calcium. To experimentally test whether PC1 binds CaM, membrane-anchored constructs containing the full-length PC1 tail (FLM), or the N-terminal (NTM), or C-terminal (CTM) halves of the PC1 tail (Fig 2A) were expressed in HEK293T cells and incubated with CaM agarose beads. As shown in Fig 2B, the full-length and N-terminal half of the PC1 tail bind strongly to CaM, whereas the C-terminal half does not. This is consistent with the location of the predicted CaM binding sites within the N-terminal half of the PC1 tail.

To test whether the binding between PC1 and CaM is dependent on Ca^2+^, similar CaM-precipitation experiments with the PC1 tail were carried out either in the absence or presence of Ca^2+^ (200 μM buffered Ca^2+^). As shown in Fig 2C, CaM binding to PC1 depends on Ca^2+^ as predicted.

CaM has four EF-hands that each bind to Ca^2+^ with different affinities. To further investigate the dependency of the PC1/CaM interaction on Ca^2+^, binding experiments were carried out with a range of free Ca^2+^ concentrations (Fig 2D and 2E). The results demonstrate that PC1-FLM binds to CaM at very low concentrations of Ca^2+^, suggesting that only the high affinity EF-hands of CaM need to be bound to Ca^2+^ for the interaction to occur.

Next, point mutations were introduced in the PC1 tail in an attempt to disrupt the interaction with CaM. Mutagenesis of the first predicted CaM binding motif alone, without altering the second predicted CaM binding motif, did not disrupt the overall ability of the PC1 tail to bind CaM (Fig 2F). In contrast, mutating the well-conserved serine (S4144) in the second domain to an aspartic acid resulted in strong attenuation of binding between CaM and the PC1 tail (Fig 2G). By sequence analysis [35], the S4144D mutation is predicted to eliminate CaM binding in the second putative CaM binding motif, presumably by reducing the basic charge that is characteristic of the 1-5-8-14 motifs (Fig 1C).

Furthermore, deletion of the first putative CaM binding motif from the PC1 tail retained binding to CaM, but this binding was largely eliminated by simultaneous mutation of serine 4144 (Fig 1H). Finally, we also found that a previously identified patient mutation that occurs within the second putative CaM-binding motif (L4137P) strongly attenuated CaM binding (Fig 2I). This mutation was previously classified as “Highly Likely Pathogenic” [37].

The PC1 constructs used in binding experiments for Fig 2G–2I are non-membrane-anchored, soluble versions of the PC1 tail that mimic the native cleavage product of PC1 termed PC1-p30. We and others have shown that PC1-p30 is overexpressed in polycystic kidneys, targets to the nucleus and regulates the activities of several transcription factors including STAT3, STAT6, TCF and CHOP [10–12, 17, 27]. Therefore, these results suggest that PC1 can interact with CaM irrespective of its proteolytic processing and membrane-anchorage.

Altogether, these results identify the second predicted CaM binding motif as the major CaM binding site in the cytoplasmic tail of PC1. The interaction is Ca^2+^-dependent, and mutation of this motif results in overall reduced CaM binding to PC1 that may be associated with disease pathogenesis. These findings led us to hypothesize that CaM binding to PC1 may be necessary for one or more PC1-mediated functions.

### Mutation of the CaM binding motif does not affect PC1’s ciliary localization nor its regulation of AP1 and STAT3 signaling

Full-length, membrane-anchored PC1 localizes to the endoplasmic reticulum [20, 38] and primary cilia [5] of renal epithelial cells [9–11, 13]. To investigate how CaM binding may affect the function or localization of PC1, we generated stable MDCK renal epithelial cell lines that express either wild-type PC1 or PC1 carrying the S->D mutation that disrupts the main CaM binding site. For these experiments, mouse PC1 was used instead of human PC1 because the available mouse expression construct results in much more robust expression. The sequences of the cytoplasmic tails are extremely conserved between mouse and human and the equivalent mutation (S4134D) was introduced to disrupt CaM binding. Expression of PC1 in these cell lines can be controlled due to the use of a doxycycline-inducible promoter.

**Fig 3:**
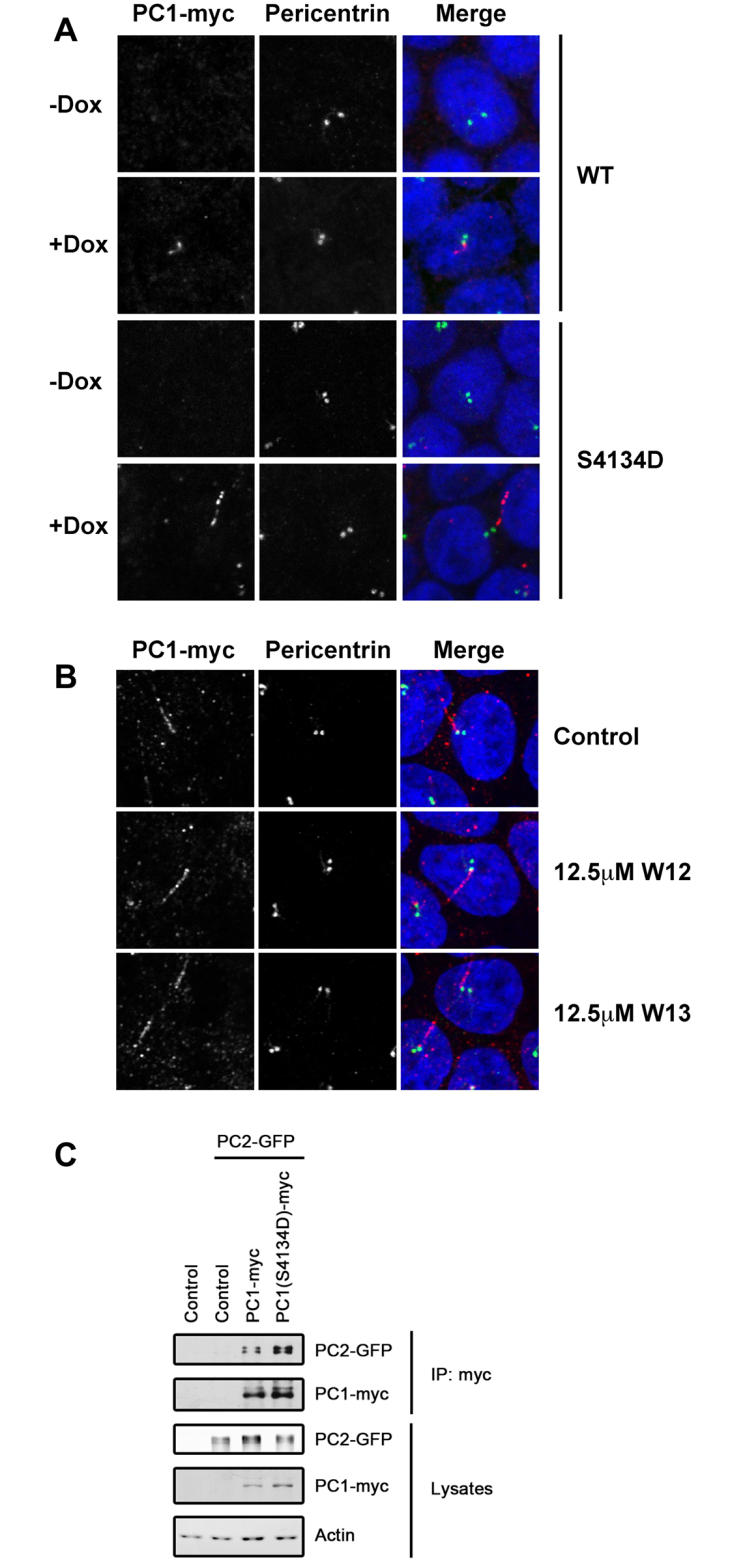
Inhibition of calmodulin does not prevent targeting of PC1 to cilia or its interaction with PC2. (A) Immunofluorescence microscopy of doxycycline (Dox)-inducible, stable MDCK cell lines expressing wild-type or mutant full-length mouse PC1 when grown in the absence or presence of 50ng/mL Dox. (B) Localization of wild-type PC1 in Dox-induced MDCK cells treated with 12.5 μM of the CaM inhibitor W13 or the control compound W12. (C) Co-immunoprecipitation of PC2 with wild-type or mutant PC1 from lysates of transiently-expressing HEK293T cells.

In fully polarized MDCK cells grown on Transwell filters, PC1 largely localized to primary cilia (Fig 3A). PC1-S4134D also localizes strongly to primary cilia, suggesting that CaM binding is not necessary for the cilia targeting of PC1. This finding was confirmed by incubating the cells with the CaM inhibitor W13 which did not affect the cilia localization of PC1 (Fig 3B).

PC1 and PC2 are known to interact via coiled-coil domains in their cytoplasmic tails [24–26, 39]. Although the CaM binding motif in PC1 is not very near the coiled-coil domain, we tested whether mutation of the CaM binding motif affects the PC1-PC2 interaction. Wild-type or S4134D-mutant full-length PC1 were co-expressed with PC2 in HEK293T cells and their interaction analyzed by co-immunoprecipitation. Both wild-type and mutant PC1 bound equally well to PC2 (Fig 3C), suggesting that CaM binding is not necessary for the PC1-PC2 interaction.

**Fig 4:**
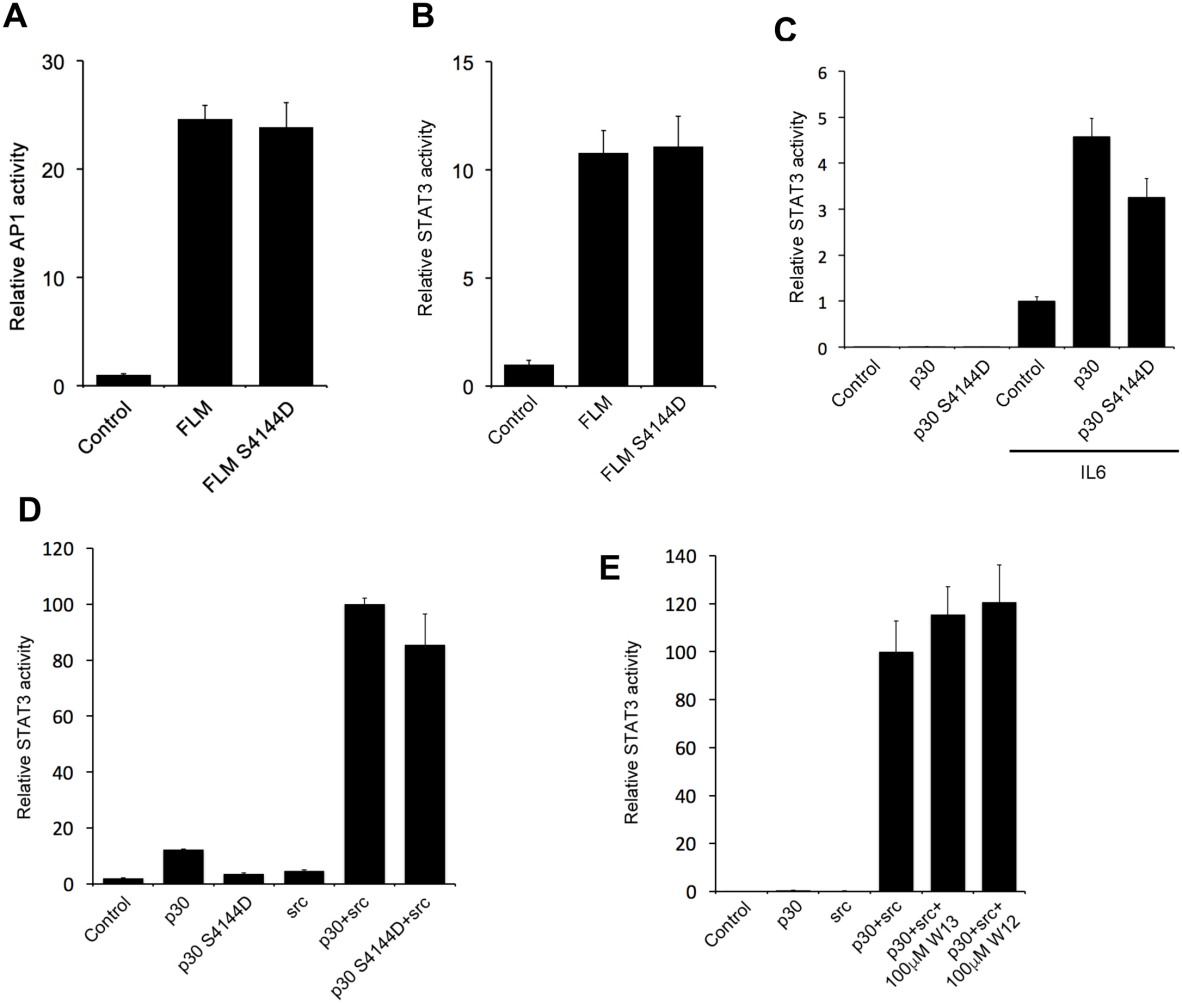
Disruption of CaM binding does not affect PC1-mediated G-protein or STAT signaling. HEK293T cells were transfected with the indicated PC1 constructs and analyzed for AP1 (A) or STAT3 (B-E) activity using the respective luciferase transcriptional reporters. Mutation of the CaM binding site in membrane-anchored FLM does not affect AP1 (A) or STAT3 activation (B). (C) PC1-p30-dependent co-activation of STAT3 in HEK293T cells expressing wild-type or mutant PC1-p30 either in the presence (right 3 bars) or absence (left 3 bars) of 25 ng/mL IL6. (D) PC1-p30- and src-dependent activation of STAT3. (E) The CaM inhibitor W13 does not inhibit the PC1-p30/src-dependent activation of STAT3.

PC1 has been reported to activate AP1/JNK signaling via activation of heterotrimeric G-proteins [9]. Importantly the region of the PC1 tail that activates heterotrimeric G-proteins [7] overlaps significantly with the CaM binding motif identified here (Fig 1C). Therefore we tested the effect of the CaM-binding mutant in an AP1 luciferase reporter assay. As shown in Fig 4A, both the wild-type and mutant constructs of the PC1 tail activate AP1 signaling equally well, suggesting that CaM binding is not necessary for PC1-mediated G-protein signaling and AP1/JNK activation.

We have previously shown that the membrane-bound PC1 tail can activate STAT3 via the tyrosine kinase JAK2 [10]. Additionally we identified patient mutations that mapped near the CaM binding site that affected this activity [10]. To test whether STAT3 activation is dependent on CaM binding, a STAT3 luciferase reporter assay was used. Both the wild-type membrane-anchored PC1 tail and the CaM-binding mutant led to similar STAT3 activation (Fig 4B) indicating that CaM binding is not required for STAT3 activation by membrane bound PC1.

We also reported that the soluble, cleaved PC1 tail (PC1-p30) can co-activate STAT3 after IL6 activation [10]. To test whether this function of PC1 is dependent on CaM binding we tested PC1-p30 and the corresponding CaM-binding mutant using a STAT3 reporter assay in cells that were stimulated with IL6 (Fig 4C). Again, both wild-type and mutant PC1-p30 co-activated STAT3 similarly indicating that this function is not dependent on CaM binding.

Additionally, we previously reported that the tyrosine kinase Src can bind PC1-p30 and activate STAT3 in a JAK2-independent manner [11]. Therefore we tested our CaM binding mutant in this functional assay as well but, again, wild-type and mutant PC1-p30 led to similar Src-dependent STAT3 activation (Fig 4D). Consistent with this, the CaM inhibitor W13 had no effect in this assay (Fig 4E).

Together, these results indicate that CaM binding to the PC1 C-terminal tail is not required for cilia targeting or the ability of PC1 to signal via AP1 or STAT3.

To identify other signaling pathways that may be regulated by CaM binding to PC1, we analyzed lysates from the DOX-inducible cells expressing either wild-type or CaM-binding mutant PC1 (S2 Fig). We found that expression of wild-type PC1 reduces phospho-S6 levels consistent with the previous finding that PC1 suppresses mTORC1 signaling [15, 40, 41]. In contrast, the mutant PC1 does not affect phospho-S6 levels suggesting that CaM binding is required for mTOR regulation by PC1. Phospho-Erk and phospho-STAT3 levels, on the other hand, do not change with PC1 expression (S2 Fig). These data suggest that CaM binding to PC1 may only regulate specific PC1-dependent signaling pathways.

### PC1/PC2-mediated channel activity is disrupted by CaM inhibition

**Fig 5:**
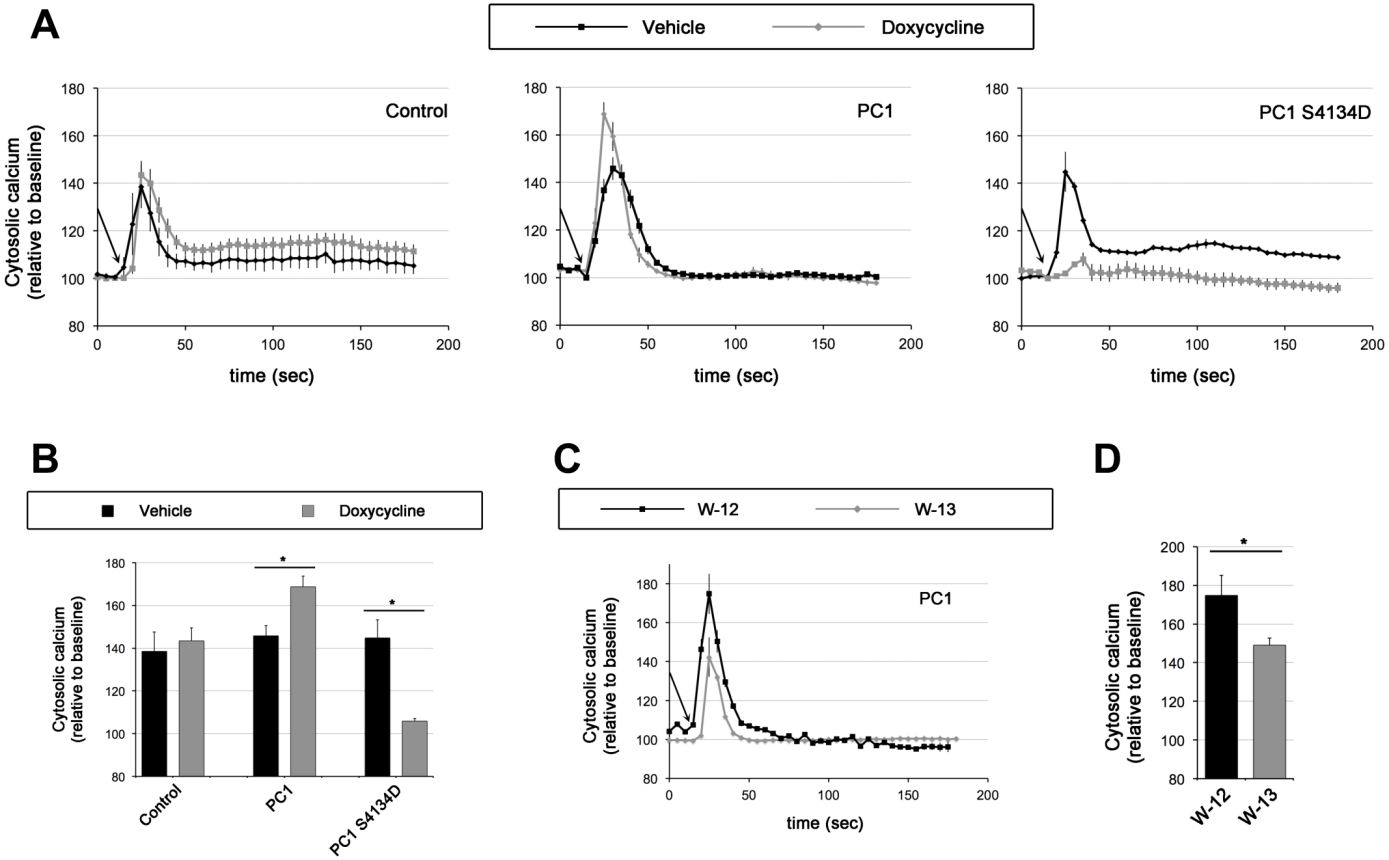
CaM binding mutants and inhibitors of CaM inhibit flow-induced calcium entry. (A) Cytosolic calcium was measured in cells treated with or without doxycycline (Dox) (50 ng/L) for 36 hours. Once the baseline calcium was stabilized, cells were challenged with fluid-shear stress. Baseline calcium was normalized to 100%, and changes in cytosolic calcium were recorded every 5 seconds (s). Arrows indicate the starts of fluid-shear stress at 1 dyne/cm^2^. (B) The maximum changes of the cytosolic calcium peak are illustrated in the bar graph. Data analysis was carried out with Prism 5 using non-parametric one-way ANOVA followed with Newman-Keuls multiple comparison post test analysis. Asterisks denote statistically significant difference between groups of cells treated with and without DOX at p<0.05. N = 5 for each group. (C) To further investigate the role of CaM on PC1 function, PC1-expressing cells treated with DOX were dosed with 12.5μM of CaM inhibitor W13 or its control analog W12. Measurements were made as in 5A. (D) The average peaks of cytosolic calcium are presented in the bar graph. Statistical analysis was done with Prism 5 using student’s t-test. N = 5 for each group, and asterisk indicates p<0.05.

PC1 is thought to function in a complex with PC2 as a mechano-sensitive Ca^2+^-permeable ion channel in the primary cilium [5, 42]. Thus, the inducible PC1-expressing MDCK cell lines were used to test the potential role of CaM-binding in flow-dependent Ca^2+^ signaling. Similar to previous reports [5] polarized MDCK cells produce a transient rise in cytosolic Ca^2+^ in response to apical fluid flow (Fig 5A). Upon doxycycline-induction to over-express wild-type PC1, MDCK cells exhibit a significantly larger Ca^2+^ increase in response to flow (Fig 5A and 5B). In contrast, MDCK cells expressing the CaM-binding mutant of PC1 exhibit a strongly suppressed Ca^2+^ increase in response to flow. This suggests that the mutant form of PC1 acts as a dominant negative inhibitor with regard to flow-induced Ca^2+^ signaling, presumably by displacing endogenous, wild-type PC1 in these cells. Given that the CaM-binding mutant of PC1 still efficiently targets to cilia (Fig 3A) and still binds to PC2 (Fig 3C) this suggests that the PC1/PC2 channel complex depends on CaM binding to mediate flow-dependent signaling.

In order to understand the dependence of this effect on flow rate, we measured calcium levels for each cell line at 0, 0.5, 1, and 8 dyne/ cm^2^ (S3 Fig). MDCK cells responded optimally to a flow rate of 1 dyne/ cm^2^. However calcium levels were elevated in wild-type PC1-expressing cells and suppressed in mutant PC1-expressing cells across all flow rates tested.

To verify that the effect is indeed due to CaM inhibition, the response of PC1-expressing MDCK cells to flow was tested in the presence of the CaM inhibitors W13 vs. the control compound W12. These inhibitors were used at levels well below their established IC_50_ concentrations to avoid any potential off-target effects. Despite this, we still noticed a significant decrease in flow-induced Ca^2+^ signaling in cells treated with W13, but not with the control compound W12 (Fig 5C and 5D). Together these data suggest that CaM binding to the PC1 tail regulates flow-induced Ca^2+^ signaling.

**Fig 6:**
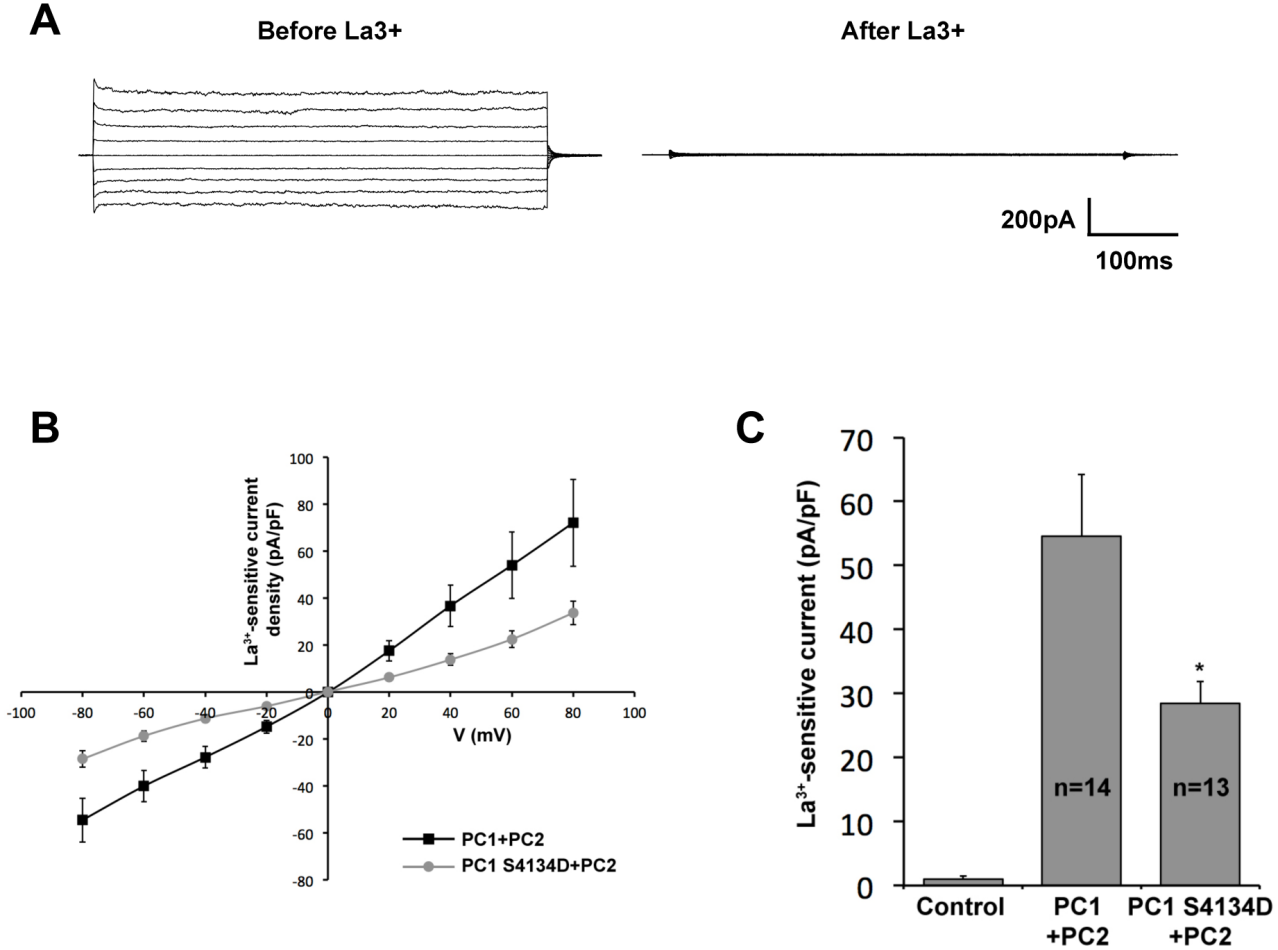
The CaM-binding mutation in PC1 decreases macroscopic current through PC1/PC2 channels. (A) Typical whole-cell currents before and after treatment with La^3+^ under voltage clamp conditions from cells transfected with wild-type PC1 and PC2. Currents were elicited by test pulses with 20 mV steps from 80 to -80 mV from a holding potential of 0 mV. (B) La^3+^-sensitive current-voltage (I-V) relation in wild type vs S4134D mutant PC1 coexpressed with PC2. (C) Summary graph of the La^3+^-sensitive current density at –80 mV. The control condition refers to non-transfected cells. The number of observations in each group is shown. Asterisk indicates *p* <0.05.

To investigate the channel activity of the PC1/PC2 complex more directly we measured La^3+^-sensitive whole cell macroscopic currents from CHO cells transiently expressing both PC1 and GFP-tagged PC2 as described previously [43]. CHO cells expressing both proteins produced strong currents that were eliminated in the presence of La^3+^ (Fig 6A). PC1 containing the CaM-binding mutation produced significantly weaker currents than wild-type PC1 (Fig 6B and 6C), suggesting that PC1’s interaction with CaM is required for full PC1/PC2 channel activity.

Altogether these results indicate that PC1-dependent channel activity is enhanced by binding of CaM to the motif identified here, and that inhibition of this interaction reduces channel activity and flow-dependent Ca^2+^ signaling.

### Expression of the CaM-binding mutant of PC1 alters cellular respiration

**Fig 7:**
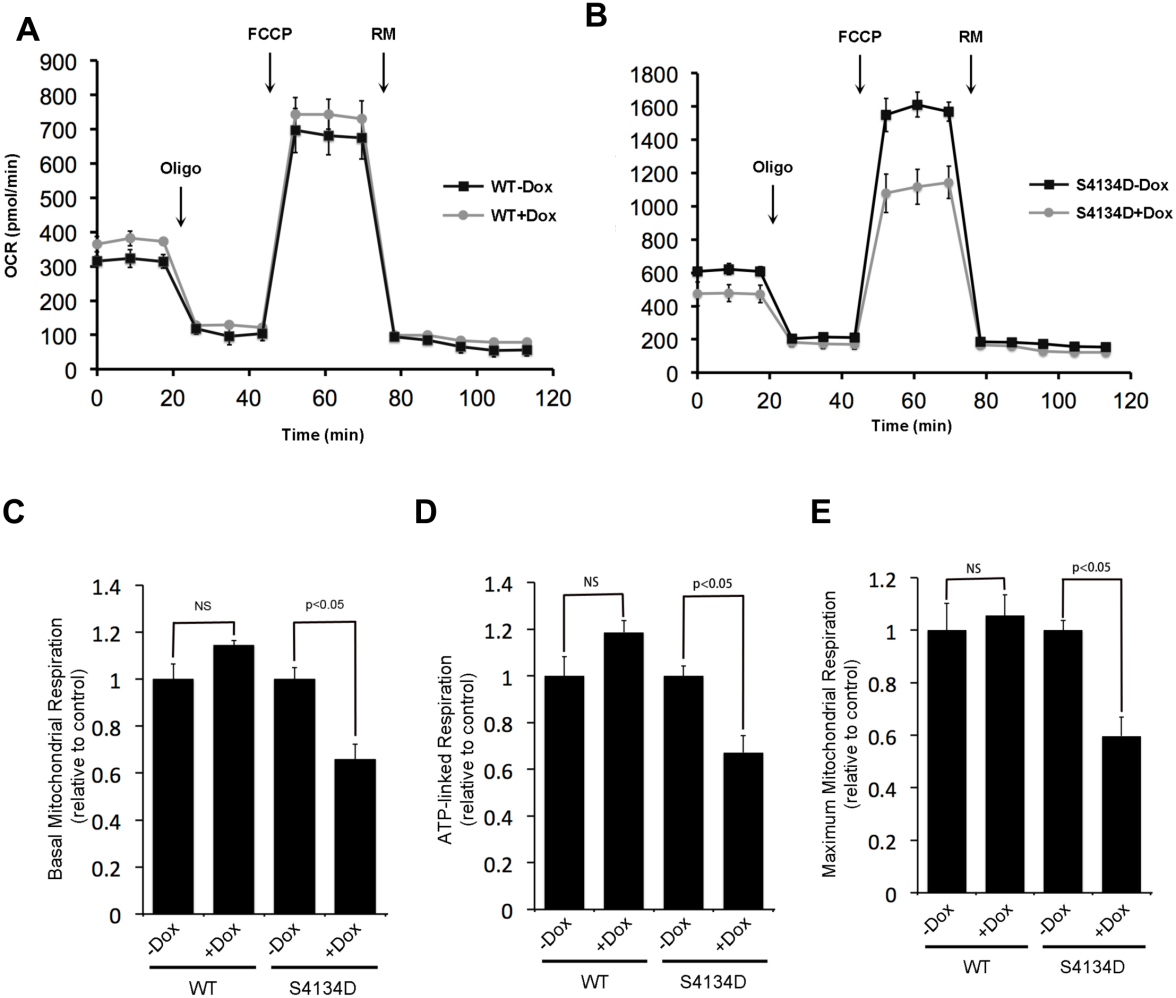
Expression of the CaM-binding mutant PC1 alters energy metabolism in MDCK cells. Oxygen consumption rate (OCR) was measured using DOX-inducible, stable MDCK cells expressing either wild-type PC1 (A) or mutant PC1 (B). OCR was measured before and after the sequential injection of 1 μM oligomycin, 0.25 μM FCCP, and 0.75 μM of rotenone. (C) Quantification of relative basal mitochondrial respiration of cells compared to uninduced controls (controls set to a value of 1). (D) Quantification of relative ATP-linked respiration compared to uninduced controls. (E) Quantification of maximum mitochondrial respiration compared to uninduced controls.

PC1 has recently been shown to regulate cell metabolism [41]. To test the potential role of CaM binding to PC1 on cell metabolism we measured the oxygen consumption rates (OCR) of DOX-inducible MDCK cells expressing wild-type or mutant PC1 using a Seahorse cell metabolism analyzer in the absence of fluid flow. Cells induced to express wild-type PC1 exhibited slightly higher, albeit not significant (p = 0.08), basal mitochondrial respiration (Fig 7A and 7C) and ATP-linked respiration (Fig 7D and S4 Fig) relative to uninduced controls. In contrast, cells expressing the CaM-binding mutant exhibited sharply lower oxygen consumption rates (Fig 7B and S4 Fig), reflecting lower basal mitochondrial respiration (Fig 7C), ATP-linked respiration (Fig 7D), and maximal mitochondrial respiration (Fig 7E).

These data support a previous report [41] that PC1 is an important regulator of cell metabolism and further suggest that CaM binding to PC1 is necessary for the normal regulation of this function. As measurements were performed in the absence of apical fluid flow, these metabolic changes are likely independent of cilia-dependent flow-sensing.

## Discussion

Here we report for the first time that the PC1 C-terminal tail has potential structural homology to the regulatory domain of myosin heavy chains and contains two predicted CaM-binding motifs. We confirm the ability of CaM to interact with the second predicted CaM-binding motif, and identify a mutant that largely disrupts this interaction.

The sequence similarity we identified between regions of PC1 and myosin led us to hypothesize that these regions may have similar interaction partners. However, it remains unclear what the evolutionary relationship is between these two proteins. As they share relatively little sequence homology outside of the identified region, it is possible that the similarity is due to convergent evolution.

PC1 is not the only ciliopathy-related protein that interacts with CaM. Nephrocystin 5 and inversin both have two IQ domains, interact with CaM, and localize to cilia [44–46]. It was also found that CaM itself localizes to cilia [44], suggesting that this is where these proteins interact. Given that bending of primary cilia results in increased cytosolic Ca^2+^ [5], CaM binding may be a general strategy to regulate signaling by ciliary proteins, and thus mutations in these regulatory regions may explain some causes of disease.

We have previously shown that a PEST motif within the C-terminal tail of PC1 regulates its protein stability [12]. Furthermore this motif has recently been shown to be necessary for calpain-mediated degradation of PC1 [47]. CaM binding domains are commonly associated with PEST sequences, however the full functional implications of this association remains unclear [48]. It is tempting to speculate that CaM binding to PC1 may regulate the stability and signaling of the polycystin complex by regulating access to the PEST domain.

We found that binding of CaM to the identified binding motif in PC1 is not required for PC1’s function in promoting STAT3 or AP1 signaling (Fig 4). This was surprising given that this region of PC1 also interacts with JAK2 [10] and heterotrimeric G-proteins [7]. We found, however, that inhibition of CaM binding results in decreased PC1/PC2 Ca^2+^ channel activity and potently inhibits flow-dependent Ca^2+^ signaling. These results suggest that CaM serves to regulate the PC1/PC2 ion channel complex. The polycystins have been found by several investigators to localize both to primary cilia and the ER. It is possible that CaM binding to PC1 in the ER may regulate different Ca^2+^-sensitive processes than it does in the primary cilium. Previously reported ER-associated functions of PC1 that may be regulated by CaM binding include regulation of store-operated Ca^2+^ entry (SOCE) [20, 49] and IP_3_ receptor-dependent Ca^2+^ release [38, 50].

We and others have also described a PC1-p30 cleavage product that translocates to the nucleus and regulates gene transcription. Although calmodulin still binds this fragment, we were unable to identify a function for this interaction. Given the accumulation of this fragment in ADPKD tissue, it remains an important question for future investigation.

The PC1-PC2 complex forms a Ca^2+^-permeable channel. Interestingly CaM has been found to bind and alter the function of several other TRP channels. The activities of TRPV4 [51], TRPM4 [52], TRPM2 [53], and TRPC6 [54] are enhanced by CaM binding. Conversely TRPC1 [55], TRPC3 [56], TRPV1 [57], TRPV5 [58], and TRPV6 [59] are inhibited by CaM. Furthermore, similar to PC1 [20], TRPC1 regulates store-operated calcium entry (SOCE) and this activity is regulated by CaM [55]. Whether CaM binding to PC1 plays a role in its regulation of SOCE remains to be investigated.

We found that expression of the CaM-binding mutant of PC1 alters cellular metabolism. This is consistent with previous reports that have shown that PC1 plays a role in regulating cellular metabolism [41, 60]. The exact mechanism through which this occurs remains poorly understood. However, our results suggest that this function requires CaM binding and likely full PC1/PC2 channel activity.

Our data are consistent with a model in which calmodulin binds PC1 in response to elevated calcium. This in turn leads to full activation of the PC1-PC2 ion channel complex, either in the cilium or elsewhere in the cell (ie the ER), which may be necessary to regulate mitochondrial-dependent metabolism. It seems likely that activation of the PC1-PC2 complex is not only a response to calmodulin binding but is also the cause, by raising local calcium concentrations sufficiently to initiate calmodulin binding. However it is also possible that other sources of calcium trigger binding of PC1 to calmodulin.

Altogether, our results add CaM as a new player to the multitude of binding partners of PC1. Given that CaM binding to PC1 is Ca^2+^ dependent, this could provide an important mechanism for regulating the functions of the polycystins.

## Materials and Methods

### Antibodies and chemicals

Anti-pericentrin antibody (cat# ab4448) was purchased from Abcam, anti-myc (clone 4A6, cat# 05–724) from Millipore, anti-GFP (clone JL-8, cat# 8371–1) from Clontech, and anti-actin (cat# A 5441) and anti-FLAG (cat# F4042) were purchased from Sigma. Anti-Erk (cat# 4696), anti-pErk (cat# 4370), anti-STAT3 (cat# 9139), anti-pSTAT3 (cat# 9145), anti-S6 (cat# 2317), and anti-pS6 (cat# 4858) were purchased from Cell Signaling. The anti-PC1 C-terminal antibody has been previously described [12]. Doxycycline was purchased from Sigma Aldrich. CaM agarose (Sepharose 4B) was purchased from GE Healthcare. Recombinant human IL6 was purchased from R&D Systems. W12-HCl was from Sigma and W13-HCl was from Tocris.

### Plasmids

The constructs for PC1-FLM, PC1-CTM, PC1-NTM, and PC1-p30 (aka PC1-FLS) have been previously described [12]. All of the other constructs of the PC1 tail were made by PCR-based Quikchange site-directed mutagenesis (Agilent Technologies, cat# 200521) from these original constructs. PC1-p30ΔC1 is a soluble construct made from a deletion of PC1-p30 and encodes amino acids Y4127-T4303 of PC1. All plasmids are made in pcDNA4/TO (Invitrogen), and empty vector was used as a negative control in calmodulin binding experiments. A PKD2 construct with an amino-terminal GFP tag was generated by the ligation of a PCR fragment that included the start codon and open reading frame of GFP in the 5’-multiple cloning region of a previously described PKD2 expression vector [24], followed by site directed mutagenesis resulting in an in-frame fusion transcript. Mutant and wild-type full-length mouse PKD1 expression constructs in a pcDNA4 backbone were made by first excising the majority of the 5’-region of PKD1 from the pcDNA3 expression construct kindly provided Stefan Somlo [61] using unique XbaI and XhoI sites and replacing it with filler DNA. The remaining 3’-PKD1 gene region was transferred into a modified pcDNA4/TO/2xMyc-6xHis (Invitrogen) backbone using the resulting unique XbaI and AgeI sites. The 2xMyc-6xHis tag was moved in frame as well as mutations in S4144 using site directed mutagenesis. The final full-length product was then reassembled using the XbaI and XhoI products from the original construct.

### Cell culture

HEK293T cells were acquired from ATCC and grown in DMEM (Corning Cellgro) + 10% FBS (Omega Scientific) + 1X Penicillin/ Streptomycin (Corning Cellgro) at 37°C in 5% CO_2_. Stable MDCK cells containing the tetracycline repressor (pcDNA6/TR) have been previously described [62]. To make inducible full-length PC1 expressing cells, we transfected these cells using the calcium phosphate method. Stable cells were selected using 300 μg/mL Zeocin and clones were selected based on their doxycycline-induced expression of the transgene. MDCK cell lines were cultured in MEM + 5% FBS + 1X Penicillin/ Streptomycin at 37°C in 5% CO2. Unless otherwise noted, expression was induced for 16 hours using 50ng/L of doxycycline diluted in growth media.

### Calmodulin binding experiments

For transient expression, HEK293T cells were plated on 6-well plates at a confluence of approximately 70% and incubated overnight to promote adherence. The next day transfection complex was made in 250 μL Optimem (Life Technologies) that contained 5 μL of Turbofect (Thermo Scientific) and 2μg of DNA per condition. Transfection complex was added to the medium and incubated with cells for an additional 16 hours.

Lysates were made from HEK293T cells expressing PC1-tail constructs by lysing them with 200 μL lysis buffer (50 mM Tris-HCl pH7.4, 100 mM KCl, 1% Triton X-100, 5 mM MgCl_2_ + protease inhibitors) containing the desired concentration of free calcium. Non-zero free calcium concentrations made by using 5 mM CaCl_2_ and varying the amount of EGTA were determined using the MaxChelator tool (http://www.stanford.edu/~cpatton/maxc.html). Conditions lacking calcium contained 5 mM EGTA only. Lysates were cleared by centrifugation at 13,000 rpm for 10 minutes. Supernatants were tumbled at 4°C with 30 μL of prewashed CaM-agarose beads for 1 hour. Precipitates were washed 3 times in lysis buffer and analyzed by immuno-blot.

### Luciferase reporter assays

The AP1 and STAT3 reporter assays were performed similarly. HEK293T cells were plated onto 12-well plates and transfected with 250 ng luciferase reporter, 20 ng β-galactosidase, and 250 ng of the gene of interest. The gene of interest for control samples was EGFP in pcDNA4. Transfection complexes were formed by combining the DNA with 2 μL Lipofectamine 2000 (Life Technologies) in 250 μL Optimem (Life Technologies). Complexes were added to cells and incubated for 4 hours. After incubation the media were changed for complete media. 25 ng/mL IL6 was added as indicated. Cells were incubated for another 16 hours lysed in 100 uL passive lysis buffer (Promega, Cat# E1941) for 30 minutes shaking at room temperature. Lysate was analyzed for luciferase activity using the Luciferase Assay System (Promega, Cat# 1501) and β-galactosidase activity. β-galactosidase activity was measured by adding 10 μL of lysate to ONPG buffer (100 mM NaPhosphate pH7.4, 1 mg/mL ONPG, 130 mM 2-mercaptoethanol, 3 mM MgCl_2_) and reading the absorbance at A405 when the samples turned light yellow. Luciferase activity values were normalized by β-galactosidase activity. Control conditions were assigned a value of one and all other values were compared to the control. For Fig 4D and 4E, STAT3 activity of PC1-p30/ Src-expressing cells was set to 100, and all other measurements were normalized to this.

### Alignments and PC1 structural modeling

Pairwise sequence alignments were performed between the sequence of the PC1 tail and proteins of known structure. A portion of the PC1 tail (4100–4158) was found to have ~25% identity and 30% similarity to the sequence of the heavy chain of the scallop myosin regulatory domain 777–836 (PDB_ID: 1SCM). Based on this alignment an approximate 3D model of the corresponding PKD1 fragment was made using programs COOT [63] and CHAIN, a modified version of FRODO [64]. The model has retained the myosin α helical fold. The N-terminal helical part of the model became slightly longer because an Ala insertion at 4112. The single amino acid deletions corresponding to a Leu and Gly in the myosin sequence were regularized with restrain to the α helical motif. Orientations of the side chains were chosen to avoid steric conflicts.

### Immunofluorescence microscopy

MDCK cells were grown on Transwell polycarbonate membranes (Corning) for 8–10 days with growth media containing serum below the filter and growth media lacking serum above the filter. Expression of PC1 was induced 16-hours prior to fixation with 50ng/L of doxycycline. Cells were fixed in -20°C methanol for 15 minutes. The methanol was rinsed 2X 2 minutes in TBS. The cells were incubated in cell block and permeabilization buffer (CBP; 2% BSA, 0.2% Triton-X100, 0.05% sodium azide in TBS) for 1 hour at 37°C. Transwells were then cut out and incubated with primary antibodies diluted in CBP overnight at 4°C. The next day they were washed 3X 5 minutes in wash buffer (0.7% fish skin gelatin, 0.05% triton X-100, in TBS) then incubated with fluorescent-conjugated secondary antibodies for 1 hour at 37°C. The Transwells were again washed 3X 5 minutes in wash buffer, followed by two rinses with TBS. The antibodies were fixed to their antigens by post-fixation for 10 minutes in 10% neutral-buffered formalin. They were then washed 2X 5 minutes in TBS and mounted onto a slide and coverslipped using Prolong Gold mounting media with DAPI (Molecular Probes). Images were taken with a Fluoview 1000 confocal laser scanning microscope. Image stacks were processed and analyzed using Image J.

### Electrophysiology

CHO (ATCC) cells were maintained with standard culture conditions (F-12K Gibco +10% FBS, 1x Gentamicin, 37°C, 5% CO2). For expression of PC1/PC2, cells were transfected with 2 μg PC1 and 1 μg PC2 plasmids per 35 mm dish using Polyfect reagent (Qiagen, Valencia, CA) according to the manufacturer’s protocol. The PC2 plasmid also expresses GFP that allowed identifying successfully transfected cells for further analysis. Whole-cell macroscopic current recordings of PC1/PC2 reconstituted CHO cells were made under voltage-clamp conditions using standard methods [43, 65]. Test pulses (500 ms each) stepping by 20 mV increments form a holding potential of 0 mV to 80 mV to -80 mV were used to generate current-voltage (I-V) relations and to measure PC1/PC2 activity at –80 mV. The pipette solution contained (in mM): 135 NaAsp, 15 NaCl, 0.2 EGTA, 0.12 CaCl_2_, 5 glucose, and 5 HEPES (pH 7.4). Cells were continuously superfused with an extracellular solution containing (in mM): NaAsp, 1 MgCl_2_, 1 CaCl_2_, 5 glucose, 5 HEPES at pH 7.4. After initial whole-cell measurements, each cell was analyzed in the presence of 0.5 mM LaCl_3_ in the extracellular solution to elicit La^3+^-sensitive current density. Current recordings were acquired with an AxoPatch 200B patch clamp amplifier (Mol. Devices, Sunnyvale, CA) interfaced via a Digidata 1440 (Mol. Devices) to PC running the pClamp 10.3 software package (Mol. Devices). All currents were filtered at 1 kHz. Whole-cell capacitance, on average 6–10 pF, was compensated. Series resistances, on average 2–5 MOhm, were also compensated.

### Measurement of flow-dependent calcium transients

After grown to confluence, stable MDCK cell lines were treated with either 1 μL PBS or doxycyline (final concentration 50 ng/L) diluted in 10 mL media for about 36 hours. Cytosolic calcium was measured using a Nikon TiE microscope, and cells were challenged with fluid-shear stress of about 1 dyne/cm^2^ as previously described [66]. Briefly, cells were loaded for 30 minutes at 37°C with 5 mM Fura-2 AM (Invitrogen, Inc.). After being washed to remove excess Fura-2 AM, cells were placed and observed under the microscope for optimal fluorescence signals. After equilibrated for 10 minutes, cells were challenged at fluid-shear stress of 0, 0.5, 0.8, 1.0 dyne/cm^2^. Pairs of intracellular calcium images were captured every five seconds at the excitation wavelengths of 340 and 380 nm and emission wavelength of 510 nm. These images were captured through Fura-2 filter kit that contains 25mm 340/380 excitation filters, a dichroic mirror, and a wide band 510 nm emission filter. For better focusing, the microscope was equipped with XY-axis motorized flat top inverted stage and Nikon automatic focusing RFA Z-axis drive. For a better controlled environment, the body of the microscope was enclosed inside a custom built chamber to control CO_2_, humidity, heat and light.

### Measurement of oxygen consumption rate (OCR) of cells by Seahorse XF24 analyzer

An XF24 analyzer (Seahorse Bioscience, North Billerica, MA, USA) was used to measure the bioenergetic function of the MDCK (PC1) or MDCK (PC1-S4134D) cells. Briefly, cells were seeded at a density of 30,000 cells per well and grown in the presence or absence of 50 ng/L doxycycline. On the day of the assay, medium was replaced by unbuffered DMEM supplemented with 1 g/L glucose. OCR was measured before and after the sequential injection of 1 μM oligomycin (ATP synthase inhibitor), 0.25 μM FCCP (mitochondrial uncoupler), and 0.75 μM of rotenone (complex 1 inhibitor). Mix, wait, and measure times were 3, 2, and 3 minutes, respectively. The mitochondrial function parameters determined were: 1) basal respiration (difference between OCR of untreated cells and cells incubated with rotenone), 2) ATP-linked respiration (difference between OCR of untreated cells and cells treated with oligomycin), 3) maximal respiration (difference between OCR of cells treated with FCCP and cells with rotenone treatment).

## Supporting Information

S1 Fig Conservation of the PC1 tail with myosin. Sequence alignment of amino acids 4092–4148 of the human PC1 tail with the regulatory domains of myosin heavy chains from the following select species (accession numbers): scallop (Mizuhopecten yessoensis, BAB40711), mouse (Mus musculus, BAA19691), nematode worm (Caenorhabditis elegans, NP_505094), alligator (Alligator mississippiensis, XP_006261644), chicken (Gallus gallus, 1301275A), cat (Felis catus, XP_011288625), bumble bee (Bombus impatiens, XP_012243982), planarian (Dugesia japonica, BAA34955), fruit fly (Drosophila melanogaster, NP_724001).
(TIF)

S2 Fig Analysis of signaling pathways in wild-type and mutant PC1-expressing cell lines. Wild-type or mutant PC1 cells were grown on transwell filters for 10 days. Where indicated, PC1 expression was induced by treatment with doxycycline 24 hours prior to lysis. Lysates were analyzed by western blot using the indicated antibodies.
(TIF)

S3 Fig Cells were challenged with various magnitudes of shear stress. (A) To understand if and how control, PC1 and PC1 S4134D cells respond to a range of fluid flow, we challenged the cells with shear stress of 0, 0.5, 1.0, 8.0 dyne/cm^2^. Arrows indicate the starts of fluid-shear stress, and dox denotes doxycycline. (B) Different magnitudes of shear stress were plotted against peaks of cytosolic calcium. As expected, optimal fluid-shear was detected around 1.0 dyne/cm^2^. N = 3 experiments in each group; for each experiment 25 cells were randomly selected and analyzed.
(TIF)

S4 Fig Comparison of OCR rate in wild-type and mutant PC1-expressing cell lines. Wild-type and mutant PC1 cells were treated with doxycycline to induce expression. OCR of each cell line was measured in the presence of the indicated compounds. To allow direct comparison, OCR of each cell line was normalized to the baseline levels of uninduced controls.
(TIF)
