# Supplementary figures and images for: Regulation of Polycystin-1 Function by Calmodulin Binding
Source: PLoS One. 2016 Aug 25;11(8):e0161525. doi: 10.1371/journal.pone.0161525 (PMC4999191; doi:10.1371/journal.pone.0161525)

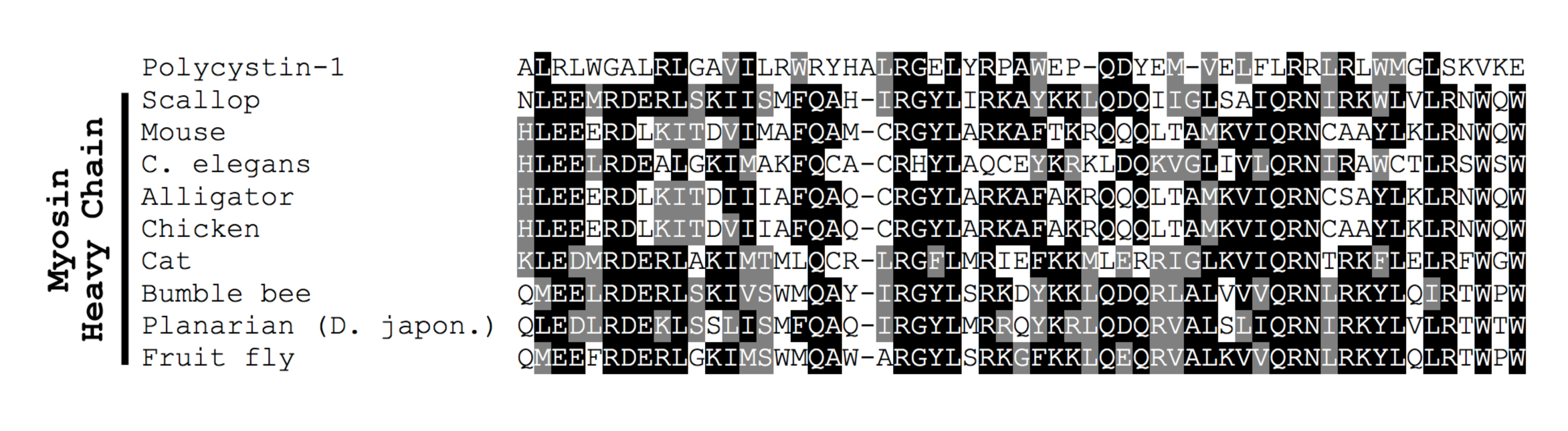

Supplement: S1 Fig — Sequence alignment of amino acids 4092–4148 of the human PC1 tail with the regulatory domains of myosin heavy chains from the following select species (accession numbers): scallop (Mizuhopecten yessoensis, BAB40711), mouse (Mus musculus, BAA19691), nematode worm (Caenorhabditis elegans, NP_505094), alligator (Alligator mississippiensis, XP_006261644), chicken (Gallus gallus, 1301275A), cat (Felis catus, XP_011288625), bumble bee (Bombus impatiens, XP_012243982), planarian (Dugesia japonica, BAA34955), fruit fly (Drosophila melanogaster, NP_724001). (TIF) [file pone.0161525.s001.tif]

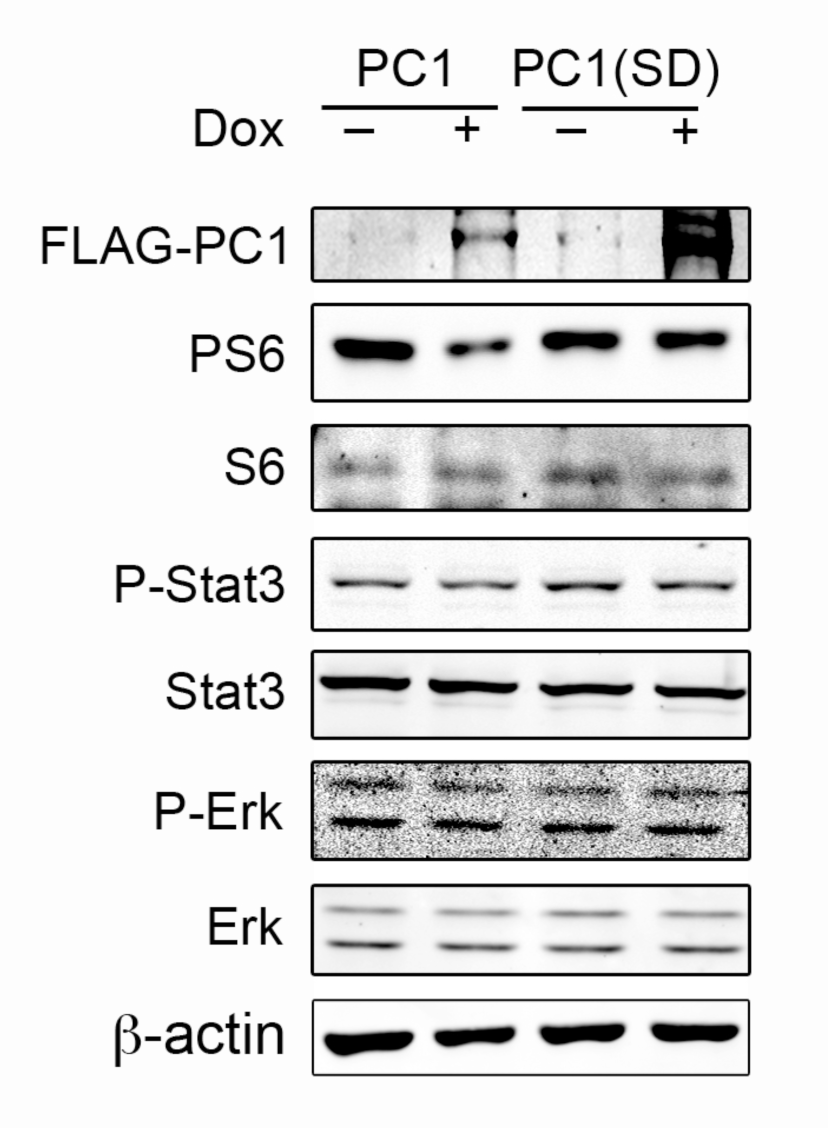

Supplement: S2 Fig — Wild-type or mutant PC1 cells were grown on transwell filters for 10 days. Where indicated, PC1 expression was induced by treatment with doxycycline 24 hours prior to lysis. Lysates were analyzed by western blot using the indicated antibodies. (TIF) [file pone.0161525.s002.tif]

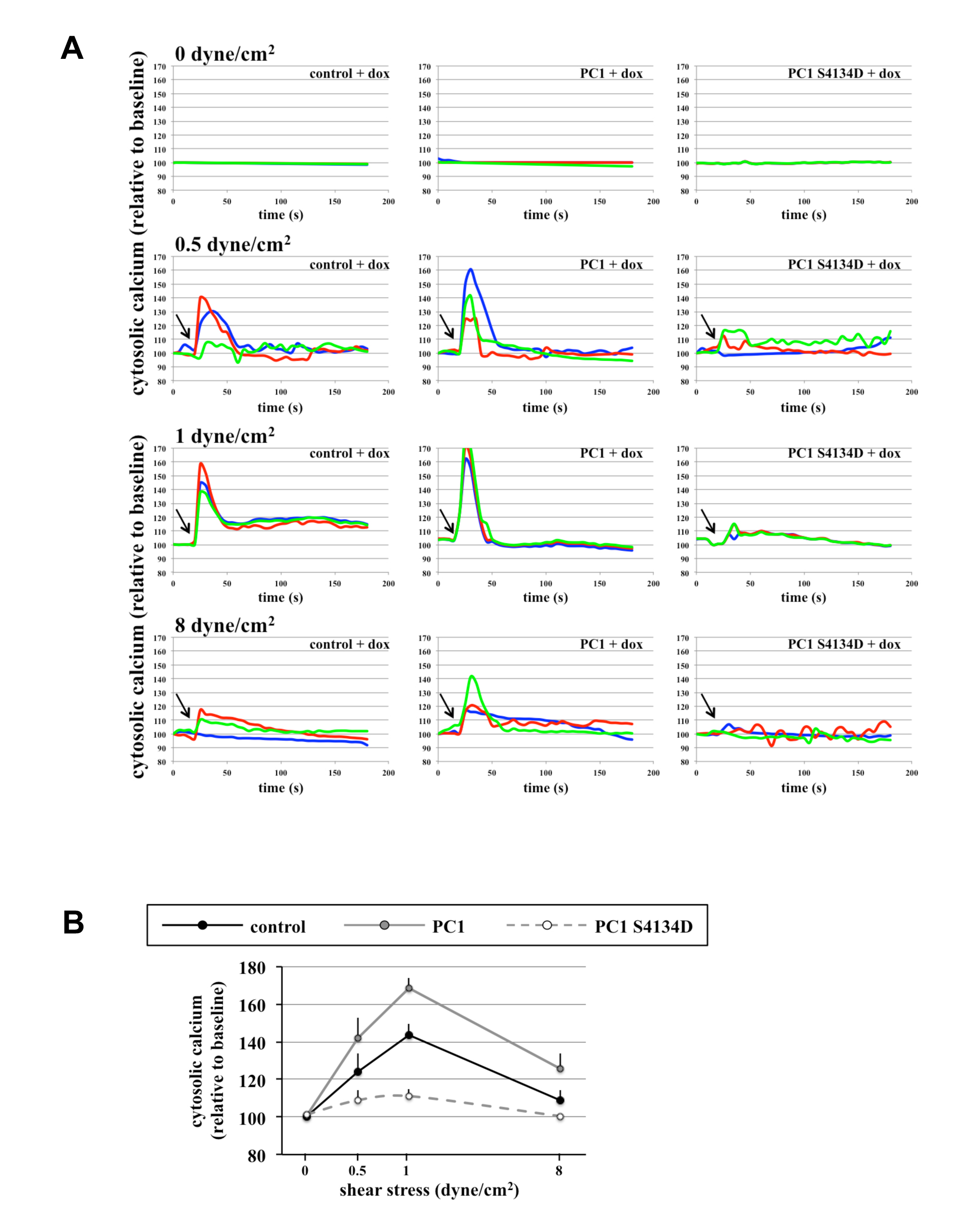

Supplement: S3 Fig — (A) To understand if and how control, PC1 and PC1 S4134D cells respond to a range of fluid flow, we challenged the cells with shear stress of 0, 0.5, 1.0, 8.0 dyne/cm2. Arrows indicate the starts of fluid-shear stress, and dox denotes doxycycline. (B) Different magnitudes of shear stress were plotted against peaks of cytosolic calcium. As expected, optimal fluid-shear was detected around 1.0 dyne/cm2. N = 3 experiments in each group; for each experiment 25 cells were randomly selected and analyzed. (TIF) [file pone.0161525.s003.tif]

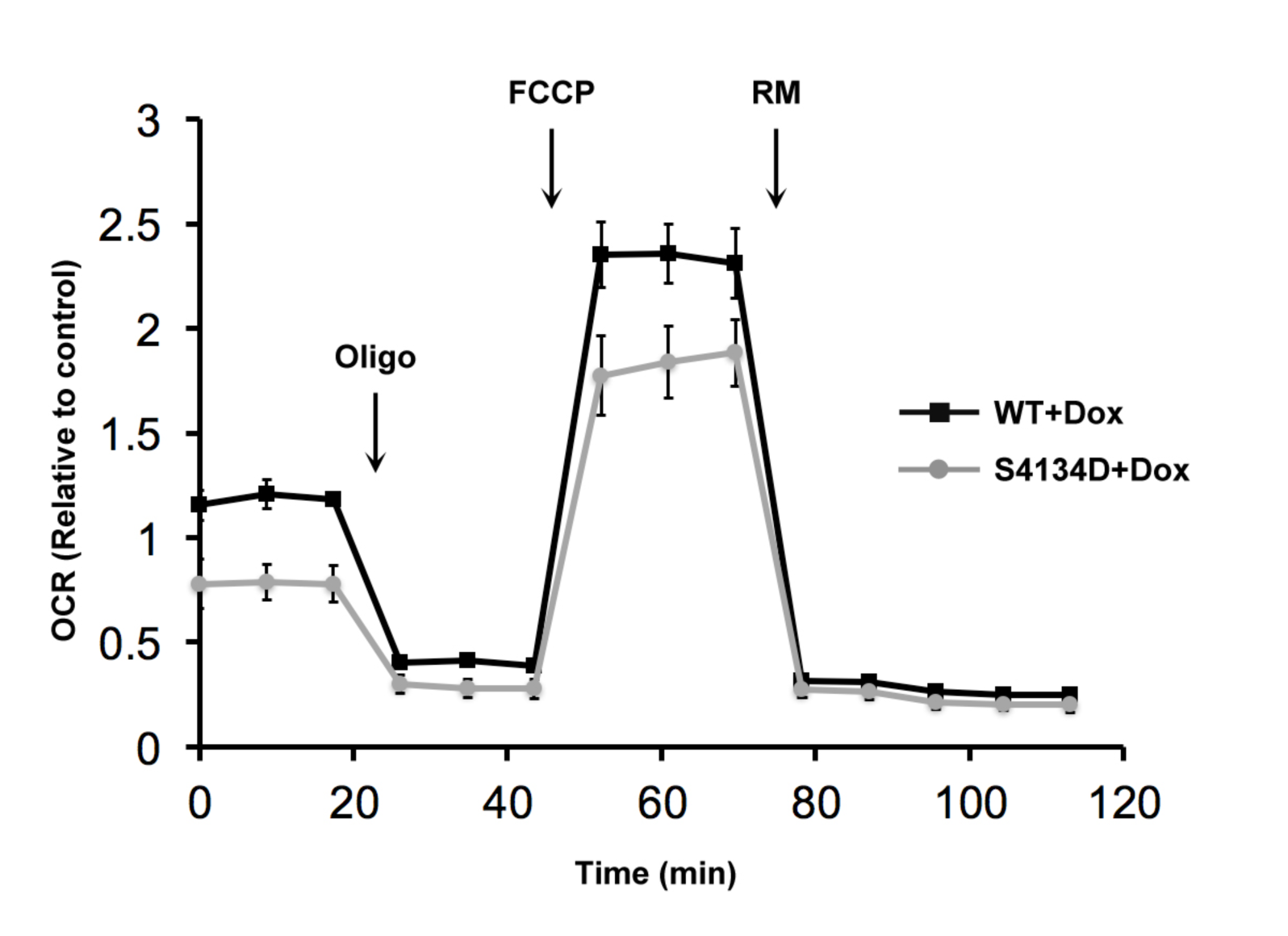

Supplement: S4 Fig — Wild-type and mutant PC1 cells were treated with doxycycline to induce expression. OCR of each cell line was measured in the presence of the indicated compounds. To allow direct comparison, OCR of each cell line was normalized to the baseline levels of uninduced controls. (TIF) [file pone.0161525.s004.tif]
